# Management of chronic knee pain caused by postsurgical or posttraumatic neuroma of the infrapatellar branch of the saphenous nerve

**DOI:** 10.1186/s13018-021-02613-0

**Published:** 2021-07-21

**Authors:** G. J. Regev, D. Ben Shabat, M. Khashan, D. Ofir, K. Salame, Y. Shapira, R. Kedem, Z. Lidar, S. Rochkind

**Affiliations:** 1 The Peripheral Nerve Reconstruction Unit, Department of Neurosurgery and Orthopedic Surgery, Tel Aviv University, Tel Aviv, Israel; 2 Sackler Faculty of Medicine, Tel Aviv University, Tel Aviv, Israel; 3 Academic Branch, Medical Corps, IDF, Tel Aviv, Israel

**Keywords:** Infrapatellar branch, Saphenous nerve, Neuroma, Neurolysis

## Abstract

**Purpose:**

Injury to the infrapatellar branch of the saphenous nerve (IBSN) is a relatively common complication after knee surgery, which can interfere with patient satisfaction and functional outcome. In some cases, injury to the IBSN can lead to formation of a painful neuroma. The purpose of this study was to report the results of surgical treatment in a series of patients with IBSN painful neuroma.

**Methods:**

We retrospectively identified 37 patients who underwent resection of IBSN painful neuroma at our institution, after failure of non-operative treatment for a minimum of 6 months. Injury to the IBSN resulted from prior orthopedic surgery, vascular surgery, tumor resection, trauma, or infection. Leg pain and health-related quality of life were measured using the numeric rating scale (NRS) and EuroQol 5 dimensions (EQ-5D) questionnaire, respectively. Clinically meaningful improvement in leg pain was defined as reduction in NRS by at least 3 points. Predictors of favorable and unfavorable surgical outcome were investigated using multivariable logistic regression analysis.

**Results:**

Patient-reported leg pain, health-related quality of life, and overall satisfaction with the surgical outcome were obtained at 94 ± 52.9 months after neuroma surgery. Postoperative patient-reported outcomes were available for 25 patients (68% of the cohort), of whom 20 patients (80.0%) reported improvement in leg pain, 17 patients (68.0%) reported clinically meaningful improvement in leg pain, and 17 patients (68%) reported improvement in health-related quality of life. The average NRS pain score improved from 9.43 ± 1.34 to 5.12 ± 3.33 (*p* < 0.01) and the average EQ-5D functional score improved from 10.48 ± 2.33 to 7.84 ± 2.19 (*p* < 0.01). Overall patient reported satisfaction with the surgical outcome was good to excellent for 18 patients (72.0%). Older age, multiple prior orthopedic knee surgeries, and failed prior attempts to resect an IBSN neuroma were associated with non-favorable surgical outcome.

**Conclusion:**

We conclude that surgical intervention is efficacious for appropriately selected patients suffering from IBSN painful neuroma.

## Introduction

**Fig. 1:**
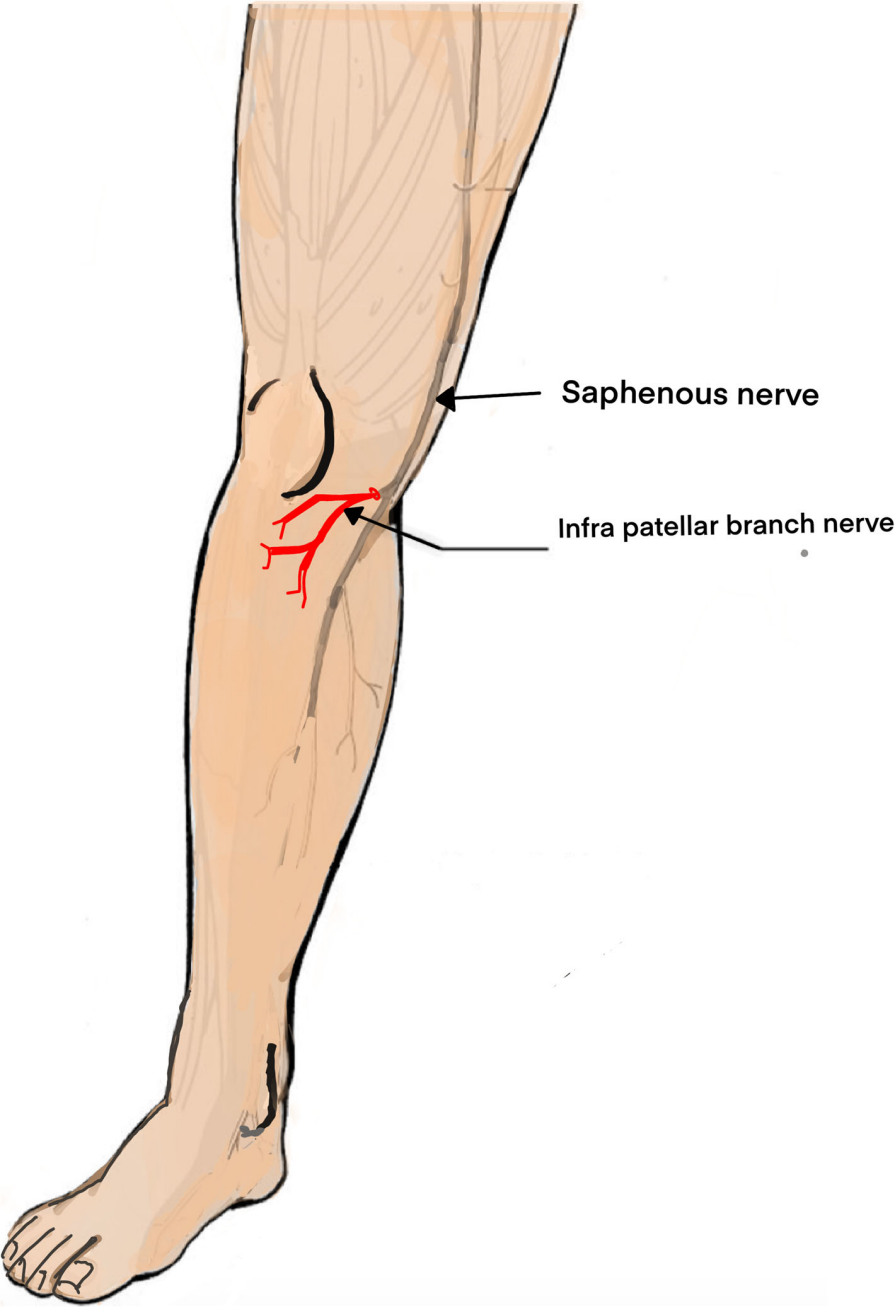
Schematic representation of the saphenous nerve and the IBSN

The infrapatellar branch of the saphenous nerve (IBSN) is a small nerve that originates at the medial aspect of the knee and provides sensation to the area of the tibial tuberosity without a motor component [1, 2]. The saphenous nerve, a branch of the femoral nerve, accompanies the femoral artery in the adductor canal. After leaving the adductor canal, the saphenous nerve gives rise to the ISBN, which emerges from the posterior aspect of the sartorius muscle at its distal end, or close to the tendons of the gracilis and semitendinosus muscles [3] (Fig. 1).

Surgical approaches to the anteromedial knee may be complicated by iatrogenic injury to the IBSN, which has been reported after total knee arthroplasty (TKA), arthroscopic knee surgery, anterior cruciate ligament reconstruction, and surgical meniscectomy [3–10]. Patients typically complain of numbness, a burning sensation, and hypersensitivity in the cutaneous distribution of the IBSN. Symptomatic IBSN injury has been reported in 55–100% of patients following TKA when a longitudinal incision is used, in 37–86% of patients following anterior cruciate ligament (ACL) reconstruction, and in up to 28% of patients following surgical meniscectomy [4, 5, 9].

Injury to the IBSN may also result in neuroma formation due to Wallerian degeneration and subsequent axonal growth [11, 12]. Due to severe and debilitating pain, many patients are limited in activities of daily living and report poor quality of life [13]. Outcomes from surgical management of painful IBSN neuroma have been previously described only in case reports and small case series [5, 6, 11, 12, 14]. In 2001, Nahabedian and coworkers reported partial or complete pain relief in 21 of 25 patients (84.0%) who underwent surgery for neuromatous knee pain and identified shorter duration of knee pain, diffuse and non-localized knee pain, and insufficient pain relief by selective nerve block as predictors of poor surgical outcome [12]. To our knowledge, these findings have not been verified in subsequent studies. More research is currently required to verify the effectiveness of surgical management and guide patient selection for surgery.

The purpose of this study was to report on postoperative outcomes from surgical neurolysis and neuroma resection in a contemporary cohort of patients with chronic knee pain due to neuroma of the IBSN and to identify prognostic factors to guide patient selection for IBSN neuroma surgery. We hypothesized that multiple prior knee surgeries, prior surgery for resection of IBSN neuroma, and higher self-reported preoperative depression or anxiety may be associated with unfavorable postoperative pain and functional outcomes.

## Methods

A retrospective review of patients who underwent resection of painful IBSN neuroma at our institution from 2001 to 2015 was performed. We included patients with painful IBSN neuroma secondary to prior surgery, trauma, or infection who had failed a trial of non-operative treatment for a minimum of 6 months.

Physical examination findings supportive of IBSN-related pain included isolated anteromedial knee pain in the distribution of the IBSN, tenderness elicited on palpation of the medial femoral condyle (Tinel sign), and associated paresthesia in the distribution of the ISBN [14]. Selective nerve block using a local anesthetic agent was performed for all patients to confirm clinical suspicion of neuropathic pain unless physical examination findings were sufficiently consistent with IBSN neuropathy such that findings from a selective nerve block would not alter the treatment plan. Sensory nerve conduction velocity (NCV) study, with or without ultrasound imaging of the knee, was obtained if diagnosis of IBSN neuropathy remained uncertain.

Preoperative leg pain and health-related quality of life were reported via a standardized questionnaire, which patients completed at a clinic visit. Postsurgical outcomes were obtained by phone interviews conducted by one of the authors (D.B.S). Patient-reported outcomes included leg pain, health-related quality of life, patient satisfaction, and sustained need for analgesic medications, physiotherapy, and/or other pain management therapies as determined by a qualified pain specialist (Table 2). Leg pain was measured using the numeric rating scale (NRS), which has been utilized in prior investigations of outcomes from surgical management of painful neuromas [15]. Clinically meaningful improvement in leg pain was defined as reduction in NRS by at least 3 points [16]. Health-related quality of life was measured using the EuroQol 5 dimensions (EQ-5D) questionnaire (Table 2), which has been validated for assessment of pain management outcomes in patients with chronic pain [17]. The minimal clinically important difference (MCID) for EQ-5D has been defined as reduction by at least 0.5 points [18]. Preoperative depression or anxiety was self-reported by patients as a component of the EQ-5D questionnaire (Table 2). Use of global patient satisfaction ratings to fully capture the physical, emotional, and psychosocial aspects of chronic pain management has been advocated in the literature [19].

### Surgical technique

An incision was made at the medial aspect of the distal thigh approximately 3 cm above the knee and carried 3 cm over the medial surface of the knee. The deep fascia to vastus medialis was opened and the muscles were retracted. Under a surgical microscope, the saphenous nerve and its infrapatellar branch were identified, and neurolysis of the infrapatellar nerve was performed. Proximal transection of the IBSN close to its origin from the saphenous nerve was performed. Marcaine 0.5% was injected into the proximal nerve stump epineurally. The proximal nerve stump was implanted firmly into an adjacent skeletal muscle and the implantation was secured with sutures. All surgeries were performed by the senior author (S.R).

### Statistical analysis

In univariable analysis, preoperative and postoperative NRS and EQ-5D scores were compared by paired *t* test parametric analysis and categorical variables were compared by Fisher’s exact test. Multivariable analysis was performed using multiple logistic regressions to identify independent predictors of postsurgical outcome. Data was analyzed using IBM SPSS Statistics for Windows, version 24.0 (SPSS Inc., Chicago, IL USA). Statistical significance was defined as *p* < 0.05.

### Ethical considerations

Approval for this research was obtained from the Institutional Research Ethics Board. All patients provided informed consent prior to enrollment in the study.

## Results

From 2001 to 2015, surgical neurolysis and IBSN neuroma resection were performed for 37 patients, including 17 females and 20 males, at our institution of whom 25 patients (67.6%) were available to answer a self-reported pain outcomes questionnaire at 94 ± 52.9 months post-surgery and 21 patients (57%) were available to answer the EQ-5D questionnaire. Average patient age was 38.9 ± 12.5 years.

Injury to the IBSN was associated with prior orthopedic surgery in 20 patients (54.1%), vascular surgery with saphenous vein harvesting or femoral-popliteal arterial graft in 3 patients (8.1%), tumor resection in 6 patients (16.2%), blunt trauma in 5 patients (13.5%), penetrating trauma in 2 patients (5.4%), and Erysipelas infection in 1 patient (2.7%) (Table 1). Prior orthopedic procedures included 21 arthroscopic knee surgeries in 13 patients, 5 reconstructions of the ACL in 5 patients of which 4 have also underwent previous arthroscopic surgery, 1 high tibial osteotomy, 1 meniscal transplantation, and 4 procedures for open reduction and internal fixation (Table 1). Tumor resection was previously performed for 2 osteochondromas and 1 benign fibrous histiocytoma of the distal femur or proximal tibial as well as 1 glomus tumor and 2 schwannomas. In addition, 3 patients reported prior unsuccessful IBSN neuroma resection (Table 1).

**Table 1:** Patient demographics, primary injury, and the number of prior surgeries at the knee area that preceded the formation of an IBSN neuroma and clinical presentation

Patient number	Gender	Age (years)	Knee arthroscopy	ACL recostruction	HTO	Trauma	ORIF	Meniscal transplant	Vascular surgery	Tumor	Infection	Prior IBSN neuroma resection
**1**	**M**	**42**	**2**									
**2**	**M**	**35**	**2**	**1**								
**3**	**M**	**59**	**1**		**1**							
**4**	**F**	**36**	**1**									
**5**	**M**	**42**	**2**									
**6**	**M**	**40**	**1**									
**7**	**F**	**48**	**2**									
**8**	**M**	**42**		**1**								
**9**	**F**	**37**	**2**									
**10**	**F**	**41**	**1**									
**11**	**M**	**44**	**1**	**1**								
**12**	**M**	**46**	**4**	**1**								
**13**	**M**	**55**	**1**					**1**				
**14**	**F**	**26**					**2**					
**15**	**F**	**27**					**1**					
**16**	**F**	**20**								**3**		
**17**	**M**	**31**							**2**			
**18**	**F**	**24**									**1**	
**19**	**F**	**61**				**1**						
**20**	**F**	**29**				**1**						
**21**	**F**	**23**				**1**				**1**		
**22**	**M**	**23**								**1**		
**23**	**M**	**50**										**3**
**24**	**F**	**51**				**1**						
**25**	**F**	**32**				**1**						**1**
**26**	**M**	**37**					**1**					**1**
**27**	**F**	**17**					**1**					
**28**	**F**	**53**							**1**			
**29**	**M**	**54**							**1**			
**30**	**F**	**18**				**1**						
**31**	**M**	**36**				**1**						
**32**	**F**	**31**	**1**									
**33**	**M**	**24**				**1**						
**34**	**M**	**31**								**1**		
**35**	**F**	**64**								**1**		
**36**	**M**	**58**								**1**		
**37**	**M**	**36**		**1**								
**Total number of patients**			**13**	**5**	1	8	4	1	3	6	1	**3**
**Total number of prior surgeries**			**21**	**5**	1	8	5	1	4	8	1	**5**

*ACL* anterior crusiate ligament, *HTO* high tibial osteotomy, *ORIF* open reduction and internal fixation

Preoperative sensory NCV study was obtained for 27 patients and conduction impairment of the saphenous nerve and IBSN was identified in 24 patients (88.9%). In addition, ultrasound imaging of the knee was obtained for 15 patients and IBSN neuroma was identified in 7 patients (46.7%). All patients underwent ambulatory surgery and were discharged from the hospital on the same day. Only 1 patient required wound revision and antibiotic treatment for postoperative wound infection. No other intraoperative or postoperative complications were reported.

Following surgery, 20 patients (80.0%) reported improvement in leg pain, 17 patients (68.0%) reported clinically meaningful improvement in leg pain, and 17 patients (68.0%) reported improvement in health-related quality of life (Table 2). The average NRS pain score improved from 9.32 ± 1.34 to 5.12 ± 3.33 (*p* < 0.01) and the average EQ-5D functional score improved from 10.47 ± 2.19 to 7.84 ± 2.33 (*p* < 0.01) (Table 2). Overall, 18 patients (72.0%) reported they were satisfied with the surgical outcome. However, 13 patients (52.0%) reported regular postoperative usage of analgesics, 6 patients (24.0%) regularly required other postoperative pain clinic treatments, and 5 patients (20.0%) underwent repeat surgical neurolysis and recurrent IBSN neuroma resection (Table 2). The time interval between the primary and the revision surgeries was 21.4 ± 17.1 months.

**Table 2:** Patients outcomes

*Surgical outcome (n = 25)*	*Preoperative*	*Postoperative*	%
*Pain NRS*	**9.32 ± 1.34**	**5.12 ± 3.33**	
*Pain treatments*			
Analgesic medication		**13**	52%
Physiotherapy		**10**	40%
Pain clinic		**6**	24%
Revision surgery		**5**	20%
*Satisfaction from surgery*			
Very satisfied		**12**	48%
Moderately satisfied		**6**	24%
Not satisfied		**7**	28%
*EQ 5D (n = 21)*			
*Mobility*			
None	**3**	**10**	
Moderate	**13**	**15**	
Severe	**4**	**0**	
*Self-care*			
None	**10**	**23**	
Moderate	**9**	**1**	
Severe	**2**	**1**	
*Usual activity*			
None	**3**	**12**	
Moderate	**10**	**10**	
Severe	**8**	**3**	
*Pain/discomfort*			
None	**0**	**5**	
Moderate	**4**	**15**	
Severe	**17**	**5**	
*Anxiety/depression*			
None	**10**	**16**	
Moderate	**6**	**6**	
Severe	**5**	**3**	
*Total EQ 5D score*	**10.47 ± 2.19**	**7.84 ± 2.33**	

**Fig. 2:**
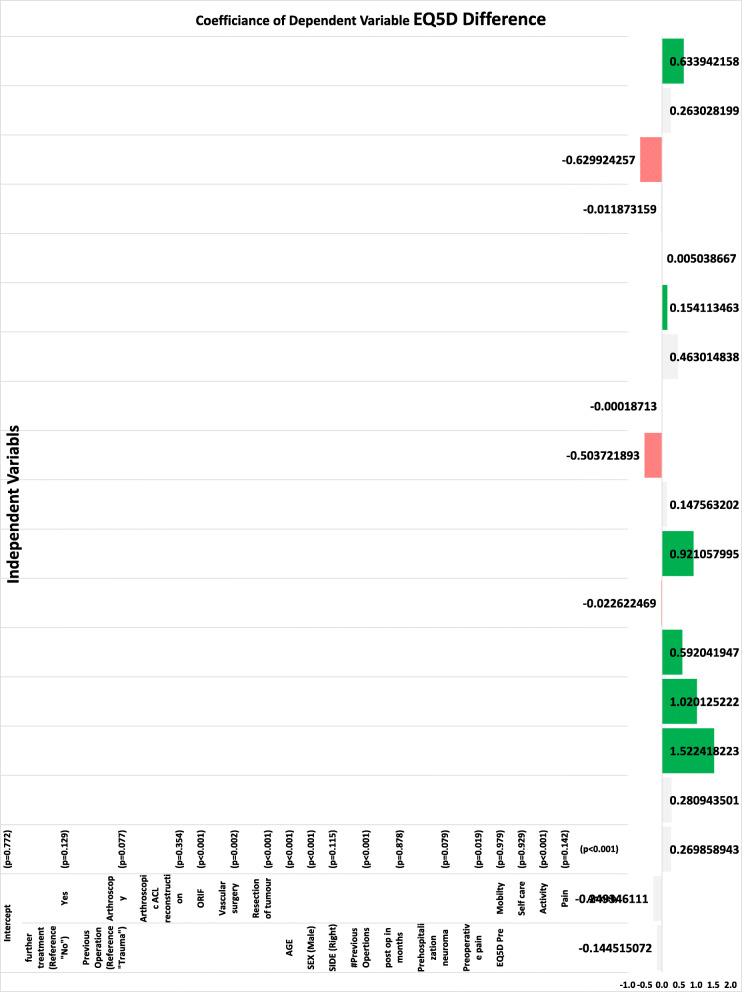
Multivariable logistic regression for independent predictors of clinically meaningful postoperative improvement in leg pain

**Fig. 3:**
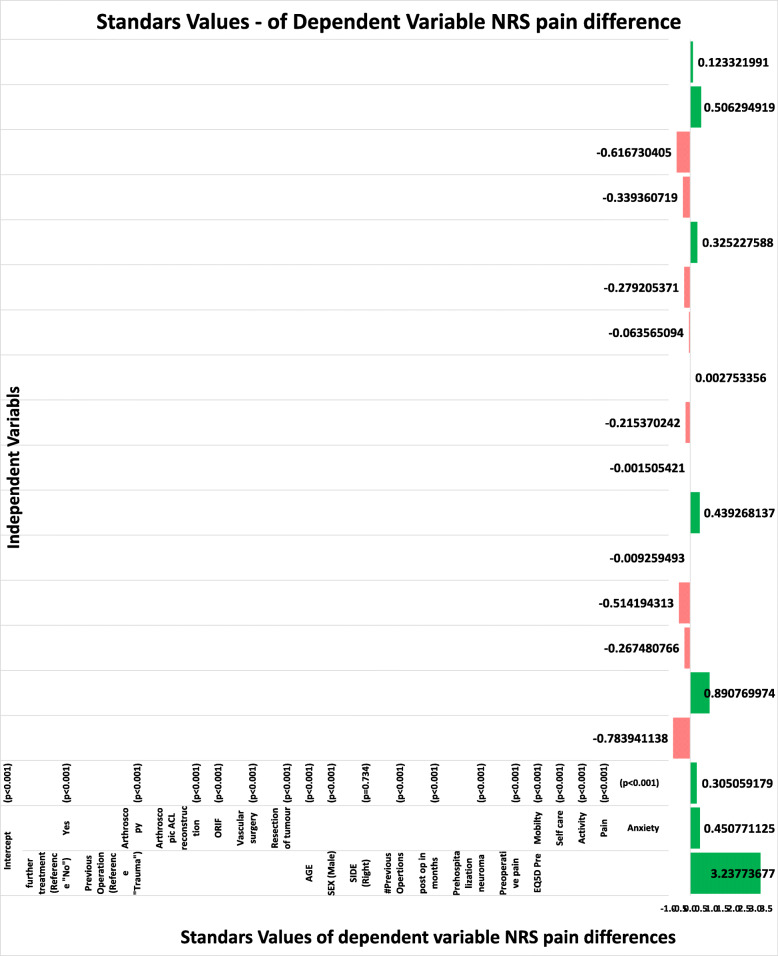
Multivariable logistic regression for independent predictors of postoperative improvement in EQ-5D

In multivariable analysis, lack of clinically meaningful postoperative improvement in leg pain was significantly associated with older age (*p* < 0.01), female gender (*p* < 0.01), multiple prior knee surgeries (*p* < 0.01), prior resection of IBSN neuroma (*p* < 0.01), lower preoperative leg pain (*p* < 0.01), lower self-reported preoperative depression or anxiety (*p* < 0.01), lower preoperative mobility (*p* < 0.01), and higher preoperative difficulty with usual activities (*p* < 0.01) and self-care (*p* < 0.01) (Fig. 2) whereas lack of postoperative improvement in health-related quality of life was significantly associated with older age (*p* < 0.01), female gender (*p* < 0.01), multiple prior knee surgeries (*p* < 0.001), lower self-reported preoperative depression or anxiety (*p* < 0.01), and higher preoperative difficulty with usual activities (*p* < 0.01) (Fig. 3).

## Discussion

In this study, we identified younger age, male gender, absence of multiple prior knee surgeries, no prior resection of IBSN neuroma, higher preoperative leg pain, and higher preoperative quality of life in the physical functioning and emotional domains as favorable prognostic factors for postoperative improvement.

Neurolysis for IBSN entrapment was first described by House and coworkers who reported excellent postoperative outcomes in 4 patients suffering from medial knee pain without prior surgery [20]. In 1996, Dellon and coworkers investigated postoperative outcomes in a cohort of 70 patients with persistent knee pain after TKA, trauma, or osteotomy and reported overall patient satisfaction and clinically meaningful improvement in leg pain for 60 patients (85.7%) [6]. A second case series was subsequently presented by Nahabedian and coworkers in 2001, in which 21 of 25 patients (84.0%) with neuromatous knee pain following TKA or trauma reported improvement in leg pain and overall satisfaction with the surgery [14].

In this study, we verified the efficacy of surgical neurolysis and neuroma resection in reducing neuromatous knee pain in a contemporary cohort of patients with IBSN neuroma secondary to various orthopedic knee surgeries, vascular surgery, tumor resection, trauma, or infection. Whereas painful neuroma of the IBSN was almost exclusively related to prior TKA or knee trauma in previous decades [5], our institutional experience suggests that arthroscopic knee surgery may be presently the most common etiology of IBSN neuropathy. In addition, we add to the existing literature by demonstrating postsurgical improvement in overall health-related quality of life in 68.0% of patients. Patients experienced statistically significant postsurgical improvement in all EQ-5D domains except for anxiety or depression (Table 2).

Patients with persistent knee pain from neuroma of the IBSN are often severely debilitated. Mean preoperative leg pain severity in our cohort was 9.32 (range, 4–10) and mean preoperative leg pain severity reported by Nahabedian and coworkers was 8.5 (range, 5–10) [14]. As such, when conservative treatments fail to provide adequate pain relief, surgical intervention should be strongly considered. However, 8 of our patients (32.0%) did not experience clinically meaningful improvement and 5 of our patients (20.0%) did not experience any postoperative improvement in leg pain. Similarly, despite stringent patient selection, Nahabedian and coworkers reported no pain relief in 4 patients (16.0%) and only partial pain relief in 10 patients (40%). In addition, mean postoperative leg pain severity was higher than mean postoperative leg pain severity in our cohort (6.7 versus 5.1). Therefore, identification of appropriate selection criteria for surgical intervention is crucial for the success of the surgical intervention.

In a prior case series, surgical intervention was only offered to patients with reduction in knee pain by at least 5 points after selective nerve block with 1% lidocaine and insufficient pain relief by selective nerve block was identified as a predictor of poor surgical outcome [14]. Surgical intervention was also offered by Dellon and coworkers only to patients with pain relief from selective nerve block [5, 6]. However, other investigators have questioned the predictive utility of selective nerve block as false-positive findings are common and may be due to the placebo effect [6, 21].

Preoperative sensory NCV studies demonstrated nerve conduction abnormality in 24 of 27 patients (88.9%) but may not be routinely available as an experienced neurophysiologist is required to perform such specific nerve conduction studies. In comparison, IBSN pathology was identified on preoperative ultrasound imaging of the knee in only 7 of 15 patients (46.7%). Future research should attempt to clarify the clinical utility of selective nerve block and sensory NCV studies in guiding patient selection for surgery.

Although mean postoperative leg pain severity remained relatively high (5.12 ± 3.33) and 5 patients (20.0%) subsequently underwent repeat surgical neurolysis and neuroma resection, 12 patients (48.0%) were very satisfied and 6 patients (24.0%) were moderately satisfied with the overall surgical outcome. Similar findings were published by Nahabedian and coworkers, who reported overall patient satisfaction in 21 of 25 patients (84.0%) despite relatively high mean postoperative leg pain severity [22]. Our findings indicate that chronic neuropathic pain is typically difficult to fully alleviate. However, patient satisfaction is influenced by multiple factors including preoperative patient expectations. In addition to appreciation of risk factors for poor postsurgical outcome, we also emphasize the need for adequate preoperative patient counseling.

This is a retrospective single institution study, which is also limited by small sample size, as IBSN neuroma is relatively uncommon and probably under-recognized and under-diagnosed. We also acknowledge potential selection bias due to loss to follow-up. However, our findings are similar to reported results from prior studies, which lend credit to overall accuracy and reliability. We await results from other contemporary cohorts to verify our current findings.

## Conclusions

Neuroma of the IBSN should be suspected in patients who develop neuropathic medial knee pain following orthopedic surgery or trauma. IBSN neuroma as a cause of debilitating chronic knee pain is likely under-recognized, particularly in community hospitals and other institutions without a dedicated peripheral nerve surgery unit. In recent years, greater emphasis has been placed on translational medicine in an effort to connect specialists to solve complex medical and surgical problems [23]. As utilization of orthopedic knee procedures continues to increase, greater prioritization of communication and collaboration between orthopedic and peripheral nerve specialists is necessary to facilitate diagnosis of postoperative neuromatous knee pain and improve clinical outcomes.

In properly diagnosed and selected patients, surgical neurolysis and resection of painful IBSN neuroma provide clinically meaningful pain improvement in a majority of patients as well as improvement in health-related quality of life. Future research should verify risk factors for poor postsurgical outcome and optimize selection criteria for surgical intervention.

## Data Availability

All data generated or analyzed during this study are included in this published article.

## Abbreviations

IBSN: Infrapatellar branch of the saphenous nerve
NCV: Nerve conduction velocity
ACL: Anterior cruciate ligament
NRS: Numeric rating scale
MCID: Minimal clinically important difference
