# Correction to: Management of chronic knee pain caused by postsurgical or posttraumatic neuroma of the infrapatellar branch of the saphenous nerve

**DOI:** 10.1186/s13018-021-02816-5

**Published:** 2021-11-23

**Authors:** G. J. Regev, D. Ben Shabat, M. Khashan, D. Ofir, K. Salame, Y. Shapira, R. Kedem, Z. Lidar, S. Rochkind

**Affiliations:** 1 The Peripheral Nerve Reconstruction Unit, Department of Neurosurgery and Orthopedic Surgery, Tel Aviv University, Tel Aviv, Israel; 2 Sackler Faculty of Medicine, Tel Aviv University, Tel Aviv, Israel; 3 Academic Branch, Medical Corps, IDF, Tel Aviv, Israel

## Correction to: Journal of Orthopaedic Surgery and Research (2021) 16:464 10.1186/s13018-021-02613-0

Following publication of the original article [1], the authors identified an error in Figs. 2 and 3. The correct figures are given below.
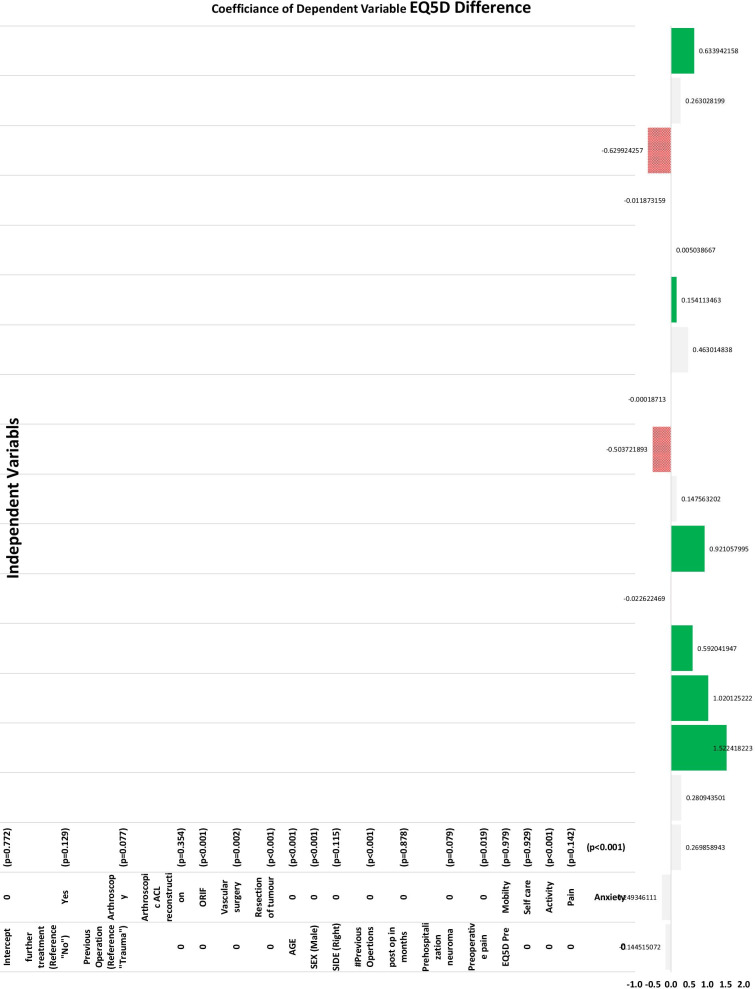
**Fig. 2** Multivariable logistic regression for independent predictors of clinically meaningful postoperative improvement in leg pain


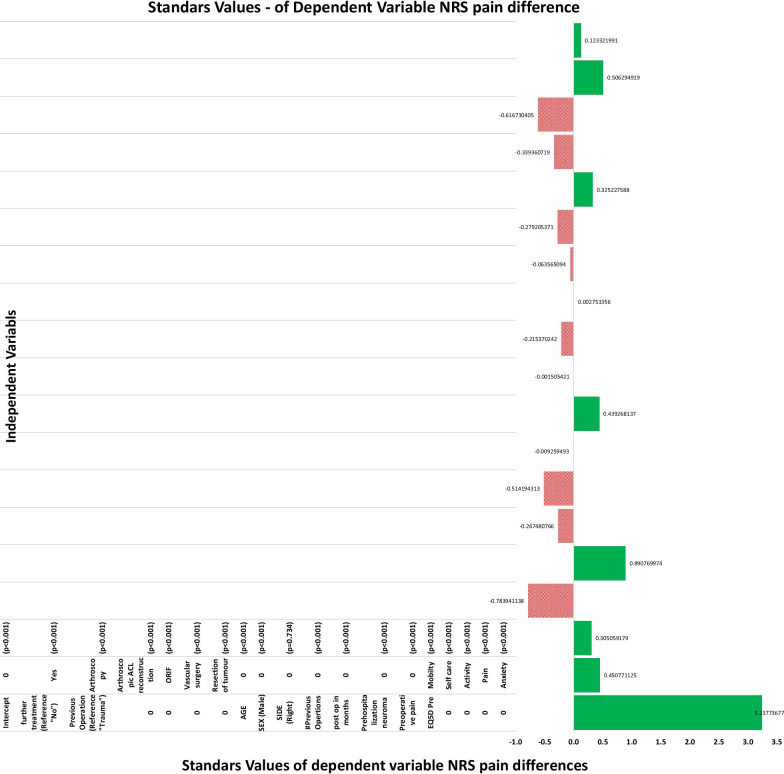
**Fig. 3** Multivariable logistic regression for independent predictors of postoperative improvement in EQ-5D

